# Biaxial Mechanical Behavior of the Choroid and Its Effect on Ocular Trauma Simulations

**DOI:** 10.1167/iovs.67.5.64

**Published:** 2026-05-26

**Authors:** Chenxi Zhang, Liping Zhang, Xiaona Li, Shaowu Sun, Nana Shangguan, Zhipeng Gao, Hongmei Guo, Zhihua Wang, Weiyi Chen

**Affiliations:** 1 Institute of Biomedical Engineering, College of Artificial Intelligence, Taiyuan University of Technology, Jinzhong, China; 2 College of Aeronautics and Astronautics, Taiyuan University of Technology, Jinzhong, China

**Keywords:** choroid, mechanical properties, finite element, impact, ocular trauma

## Abstract

**Purpose:**

Ocular trauma can cause blinding conditions such as choroidal rupture (CR) and retinal detachment (RD). The finite element method (FEM) is widely used to evaluate ocular trauma, but the choroid is often oversimplified because of limited mechanical data, potentially reducing simulation accuracy. This study characterized choroidal mechanical properties and incorporated precise viscoelastic data into ocular trauma simulations.

**Methods:**

The elastic and viscoelastic properties of the choroid were quantified using biaxial tensile testing. These data were integrated into a finite element model of the human eye incorporating the viscoelastic choroid. BB gun projectile impact simulations were then performed at different speeds to analyze the mechanical responses of posterior ocular tissues.

**Results:**

The principal constitutive parameters of the choroid were determined under equibiaxial loading. Simulation results demonstrated that, under identical ocular trauma conditions, inclusion of the choroid altered both scleral and retinal stresses compared with the choroid-excluded eye model, with retinal stress showing a larger change. At an impact speed of 60 m/s, the retinal stress change exceeded 43%. Moreover, the difference in maximum principal stress between the viscoelastic and linear elastic choroid models was 21%, 36%, and 70% at 20, 40, and 60 m/s, respectively.

**Conclusions:**

This study provides valuable reference data and a theoretical foundation for understanding the mechanical behavior of the choroid. The proposed computational model underscores the importance of viscoelastic choroid and contributes to improving the accuracy of ocular trauma modeling.

Ocular trauma is a leading cause of visual impairment and blindness worldwide, affecting millions of people each year.^1^^–^^3^ Blunt ocular trauma, the most common form of eye injury (55%),^4^ frequently occurs in accidental circumstances, with young people participating in high-impact sports (e.g., boxing, baseball, and diving) being at particularly high risk. Statistically, choroidal rupture and retinal detachment occur in approximately 5% to 10% of patients with ocular trauma.^5^^–^^7^ The choroid, a connective tissue located between the retina and sclera, is rich in blood vessels and pigmentation.^8^

Investigation of the mechanical properties of the choroid is critical for elucidating trauma-induced choroidal injury. Such mechanical damage may result in choroidal rupture and subsequently contribute to retinal detachment and degeneration.^9^^–^^11^ Numerous studies have investigated the mechanical properties of the choroid, with particular attention to its elastic^11^^,^^12^ and viscoelastic behavior.^13^^,^^14^ However, a major limitation of these studies is that the experimental data were primarily obtained from uniaxial tensile tests. Biaxial testing is more suitable than uniaxial loading for the mechanical characterization of the choroidal tissue, as collagen fibers within the choroid are predominantly aligned tangentially to the ocular surface and the tissue is primarily subjected to multiaxial loading.^15^ Biaxial testing has been widely applied to ocular tissues such as the sclera and cornea to capture their multiaxial mechanical behavior.^16^ In contrast, the biaxial mechanical behavior of the choroid remains insufficiently investigated.^17^

Finite element (FE) simulation has proven to be an effective tool for investigating blunt ocular injuries.^18^ Uchio et al.^19^ pioneered the development of a finite element eye model for dynamic analysis, investigating eye injuries caused by bullet impacts under various mechanical conditions. Stitzel et al.^20^ introduced the Virginia Tech-Wake Forest University (VT-WFU) eye model, which was validated through matched experiments and shown to accurately predict eye rupture. Previous simulation studies have involved various ocular injuries, including globe rupture,^20^^,^^21^ optic nerve damage,^22^ lens injury,^23^^,^^24^ retinal damage,^25^ traumatic retinal detachment,^26^ and primary blast ocular injuries.^21^^,^^27^ However, many ocular models have omitted the choroid, a crucial intraocular anatomical structure, to simplify the computational process.^26^^,^^28^ Excluding the choroid from the model may cause a mismatch at the retina-sclera interface, affecting stress distribution, strain transfer, and load distribution, which could reduce the accuracy of the simulation results. Furthermore, although the choroid has been incorporated into some eye models, it is often modeled with linear elasticity.^29^^,^^30^ Research has shown that the choroid is a time-dependent tissue, and it has been suggested that it should be modeled as a viscoelastic material to more realistically reflect its behavior in simulations.^10^ Therefore, ignoring or simplifying the choroid in eye modeling may affect the accuracy of model predictions.

In this study, we investigated the biaxial biomechanical behavior of the porcine choroid and obtained its elastic and viscoelastic parameters. Based on the experimental data, we developed a finite element model of ocular trauma that integrates the eyeball with a viscoelastic choroid, intraorbital fat, the orbit, and a BB gun projectile. This work not only provides experimental data to support ocular modeling but also contributes to a more accurate predictive model for ocular trauma.

## Materials and Methods

### Specimen Preparation

The 30 natural porcine eyes, aged between 6 and 8 months, were consecutively collected from a local abattoir. The eyes were immediately transported to the Laboratory of Soft Tissue Biomechanics at Taiyuan University of Technology within 2 hours to 3 hours post-slaughter. During transport, the eyes were placed in a glass container filled with 0.9% saline, and the container was then placed in a foam chamber packed with ice to prevent dehydration and tissue degradation.^31^ All animal procedures were conducted in accordance with the ARVO Statement for the Use of Animals in Ophthalmic and Vision Research, and approved by the Animal Care and Ethics Committee of the Taiyuan University of Technology (TYUT-202201100).

Before testing, the extraocular muscles, fat, and conjunctiva were carefully removed using a scalpel and forceps. The eye was then incised along the ora serrata, and the vitreous was gently decanted from the eye cup. The choroidal tissue (Bruch's membrane-choroid complex [BMCC]) was carefully isolated with minimal disruption to the surrounding posterior tissues. Prior to the tests, the thickness of the choroid specimens at the center was measured three times using a laser displacement sensor (LK-H050, Keyence Corporation), with an average thickness recorded as 0.16 ± 0.06 mm. To eliminate variations in specimen size, a consistent testing square region (8.0 ± 0.8 mm) was used. The tissue was kept hydrated with 0.9% saline throughout the preparation and testing process.

### Mechanical Testing

**Figure 1.**
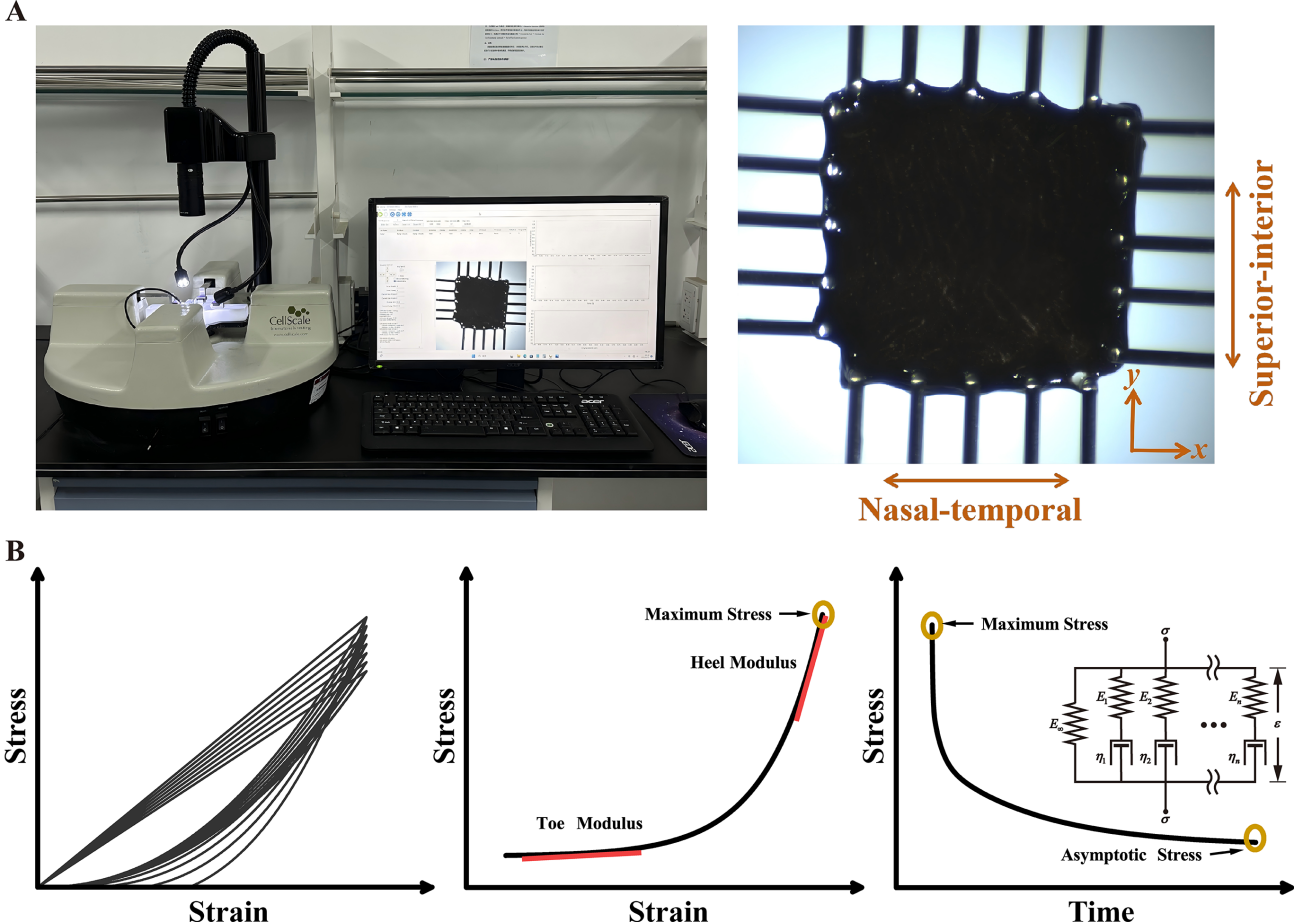
(**A**) Experimental setup for biaxial tensile testing and a representative choroidal specimen. (**B**) Representative preconditioning, stress-strain, and stress-relaxation curves of the choroid.

To simulate physiological conditions and prevent dehydration, the prepared specimens were placed in a 0.9% saline bath maintained at 37°C. A commercial biaxial testing machine equipped with 5 N load cells (BioTester, CellScale Biomaterials Testing, Waterloo, Canada) was used to perform displacement-controlled equibiaxial planar tests up to 20% strain (Fig. 1A). The device was fitted with 4 sets of rake fixtures, each consisting of 5 tines with a spacing of 1.3 mm. Choroidal specimens were tested along both the horizontal (nasal-temporal) and vertical (superior-inferior) directions. Under quasi-static conditions, 7 loading-unloading preconditioning cycles were performed to a strain of 5% at a rate of 1 mm/min.^32^ This preconditioning protocol was used to minimize hysteresis and to obtain a repeatable mechanical response. After preconditioning, the specimens were loaded to a strain of 20%. This strain level was selected on the basis of our preliminary simulations, which showed that the maximum principal strain in the choroid reached approximately 20% at an impact speed of 40 m/s. The specimens were then held at this strain for 1800 seconds to allow stress relaxation and to capture the long-term viscoelastic response until the stress approached a quasi-equilibrium state.^33^ Test data were automatically recorded and stored by the testing system.

### Data Analysis

The elastic and viscoelastic properties of the choroid were evaluated using several key parameters. The engineering stress was calculated based on the specimen thickness, the effective testing area between the rakes, and the measured force. Strain was defined as the ratio of specimen elongation to its initial length. Representative stress-strain and stress-relaxation responses (Fig. 1B) were plotted and used to determine toe modulus, heel modulus, maximum stress, and stress relaxation. The toe and heel moduli were defined as the slopes obtained from linear fitting of the stress-strain curve over the initial 2% strain interval and the final 2% strain interval prior to the maximum applied strain, respectively. Maximum stress was defined as the stress at the peak of the loading where the maximum strain (20%) was applied. The percentage of stress relaxation was calculated as the difference between the maximum and asymptotic stress values at the start and end of the 1800-second hold, which was then normalized by the maximum stress.^34^ The anisotropic index, representing directional dependency, was defined as the ratio of horizontal to vertical values and was analyzed for each parameter. A value of 1 represents isotropic behavior, whereas values deviating from 1 reflected anisotropic behavior.^35^^,^^36^

#### Passive Formulation

The Fung constitutive model incorporates both linear and nonlinear terms in the strain energy function and is widely used to describe the mechanical behavior of soft biological tissues, capturing anisotropic and nonlinear responses under different deformation conditions. In the present study, the Fung strain energy function *W*^37^ was adopted to characterize the two-dimensional stress-strain behavior of the soft tissue and can be expressed as an exponential function of the Green strain as follows in Equations 1 and 2:

(1) $$\begin{array}{c} W=\frac{c}{2}\left(e^{Q}-1\right) \end{array}$$

(2) $$\begin{array}{c} Q=c_{xx}E_{xx}^{2}+2c_{xy}E_{xx}E_{yy}+c_{yy}E_{yy}^{2} \end{array}$$

The subscripts *x* and *y* represent the horizontal and vertical directions, respectively, where *c* and *c_ij_*, *i*, *j* = *x*, *y* are coefficients in the model. Here, *c* is a stress-like material parameter; *Q* is a polynomial function of the components of *E_ij_*; and *c_ij_* are the non-dimensional material parameters in the Fung model. Specifically, the parameters *c_xx_* and *c_yy_* quantify the material stiffness in the normal strain directions, whereas *c_xy_* represents the coupling between the two directions and may be used to measure tissue anisotropy.^37^ Therefore, the ratio *c_xx_*/*c_yy_* was used as an indicator of anisotropic response in the specimens. These parameters were obtained through an optimization procedure implemented in MATLAB.

The second Piola-Kirchhoff stress components *S_ij_* can be expressed as partial derivatives of *W*, as shown in Equations 3, 4, 5, and 6:

(3) $$\begin{array}{c} S_{ij}=\frac{\partial W}{\partial E_{ij}} \end{array}$$

(4) $$\begin{array}{c} S_{xx}=ce^{Q}\left(c_{xx}E_{xx}+c_{xy}E_{yy}\right) \end{array}$$

(5) $$\begin{array}{c} S_{yy}=ce^{Q}\left(c_{xy}E_{xx}+c_{yy}E_{yy}\right) \end{array}$$

(6) $$\begin{array}{c} S_{ij}=\frac{\rho_{0}}{\rho }\frac{1}{\lambda^{2}}T_{ij} \end{array}$$

For an incompressible material, ρ_0_/ρ = 1.^37^

Based on this model, the Cauchy stresses are expressed as follows in Equations 7 and 8:

(7) $$\begin{array}{c} T_{xx}=\lambda_{x}^{2}ce^{Q}\left(c_{xx}E_{xx}+c_{xy}E_{yy}\right) \end{array}$$

(8) $$\begin{array}{c} T_{yy}=\lambda_{y}^{2}ce^{Q}\left(c_{xy}E_{xx}+c_{yy}E_{yy}\right) \end{array}$$

The components of the Green strain (*E*) were calculated using the following Equations 9 and 10:

(9) $$\begin{array}{c} E_{xx}=\frac{1}{2}\left(\lambda_{x}^{2}-1\right) \end{array}$$

(10) $$\begin{array}{c} E_{yy}=\frac{1}{2}\left(\lambda_{y}^{2}-1\right) \end{array}$$

where λ = *l*/*l*_0_ represents the ratio of the deformed length (*l*) to the resting tissue length after preconditioning (*l*_0_). Planar forces (*f*) measured by the load cells during deformation were converted to Cauchy stresses (*T*) in the principal directions, given by Equations 11 and 12:

(11) $$\begin{array}{c} T_{xx}=\lambda_{x}\frac{f_{x}}{tl_{y}} \end{array}$$

(12) $$\begin{array}{c} T_{yy}=\lambda_{y}\frac{f_{y}}{tl_{x}} \end{array}$$

where *t* is the tissue thickness.

The nonlinear regression Levenberg-Marquardt least squares algorithm in MATLAB (version 7.0.1) was used to fit the experimentally measured stresses to the corresponding theoretically calculated stresses (see Equations 7, 8) for the choroid. This optimization method is iterative, minimizing the difference between the experimentally obtained and calculated Cauchy stresses. Convexity of the function is crucial for solving numerical problems in biomechanics using the strain energy function.^35^^,^^38^ For choroidal tissues, the optimization of coefficients must be further constrained to ensure convexity. Specifically, when *c* > 0, convexity is guaranteed only if the following conditions are satisfied:

$$c_{xx}>0,\;c_{yy}>0,\;c_{xx}c_{yy}>c_{xy}^{2}$$

The coefficient of determination (*R²*), which quantifies the strength of the correlation between two variables, was calculated for each model. The set of coefficients corresponding to the highest *R²* value was selected to represent the tissue.

#### Viscoelastic Formulation

Most ocular tissues, including the choroid, exhibit viscoelastic properties. In this study, the Wiechert model was selected to characterize the viscoelastic behavior of the choroid. This model utilizes a linear combination of multiple spring-damper units to separate the transient elastic behavior from the long-term viscoelastic response (see Fig. 1B), providing an accurate representation of stress relaxation and facilitating finite element modeling.^39^

When simulated in Abaqus, the Prony series formula is utilized to assign viscoelastic properties to the material, incorporating multiple characteristic times τ_*i*_ (*i* = 1, 2, ...,  *N*). In this formulation, the time-dependent relaxation modulus *E*(t) is expressed through the Prony series^40^, as shown in Equation 13:

(13) $$\begin{array}{c} E\left(t\right)=E_{\infty }\frac{1-\sum_{i=1}^{n}m_{i}\left(1-e^{-t/\tau_{i}}\right)}{1-\sum_{i=1}^{n}m_{i}} \end{array}$$

The time-dependent relaxation modulus *E*(*t*), normalized by the initial modulus $E_{0}=E_{\infty }/\left(1-\sum_{i\;=\;1}^{n}m_{i}\right)$, can be expressed using the Prony series as $E\left(t\right)/E_{0}=1-\sum_{i\;=\;1}^{n}m_{i}\left(1-e^{-t/\tau_{i}}\right)$.

Here, *g_i_* and *k_i_* represent the normalized Prony coefficients for shear and volumetric behaviors, respectively. In Abaqus, it is assumed that *g_i_* and *k_i_* are independent of each other and can be fitted using the formulas shown in Equations 14 and 15:

(14) $$\begin{array}{c} G\left(t\right)/G_{0}=1-\sum_{i\;=\;1}^{n}g_{i}\left(1-e^{-t/\tau_{i}}\right) \end{array}$$

(15) $$\begin{array}{c} K\left(t\right)/K_{0}=1-\sum_{i\;=\;1}^{n}k_{i}\left(1-e^{-t/\tau_{i}}\right) \end{array}$$

Additionally, the volumetric parameter *k_i_* was omitted in this study, as volumetric changes of the material were not considered.^39^^,^^41^ The specific values for *g_i_* and τ_*i*_ are provided in Table 1. The instantaneous modulus *E*_0_ was calculated as the ratio of the maximum stress to the corresponding tensile strain. These parameters (*E*_0_, *g*_1_, *g*_2_, *g*_3_, τ_1_, τ_2_, *and* τ_3_) were calculated as the average of the values obtained from both directions. These averaged parameters were then implemented in the finite element model to describe the viscoelastic response of the choroid.

### Numerical Analysis

#### Eye Modeling

**Figure 2.**
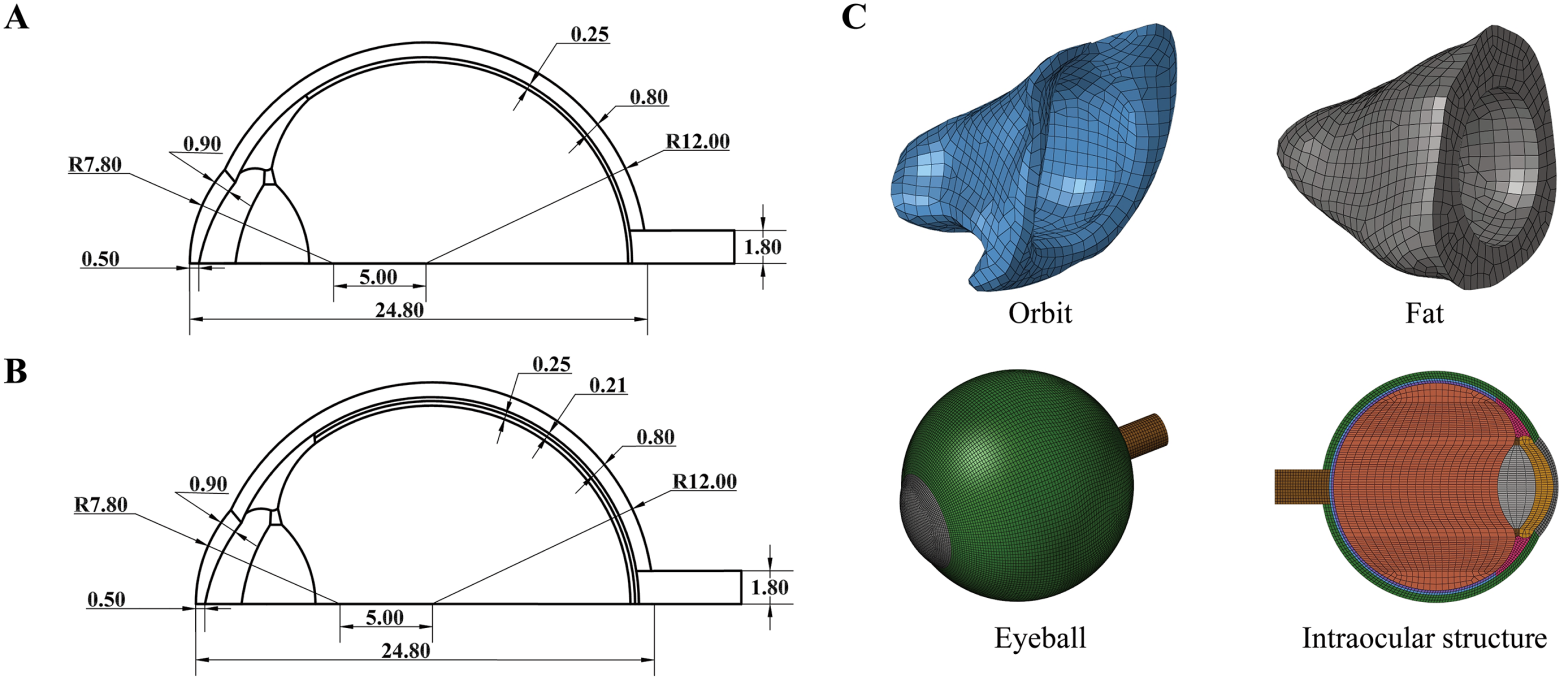
Eye model geometry. (**A**) Structure and dimensions of the eye without the choroid in cross section. (**B**) Structure and dimension of the eye containing the choroid in cross section. (**C**) Finite element model of the human eye including the eyeball model, fat model, and orbital model.

The ocular model was created using SolidWorks 2023 (Dassault Systèmes SolidWorks Corp, SA), based on the classic VT-WFU ocular model,^20^ with additional retinal and choroidal structures incorporated. A choroidal layer, with a uniform thickness of 0.21 mm,^42^ was modeled between the sclera and retina, with the retina itself having a thickness of 0.25 mm. The optic nerve head (ONH) was assumed to be located at the posterior pole of the globe. A cross-sectional view of the ocular model is shown in Figures 2A and 2B.

#### Orbital Modeling

Sequences of X-ray computed tomography (CT; 1 mm contiguous slicing) images of the human orbit were obtained from a healthy adult (aged 20 years). Informed consent was obtained from the subject, and the study adhered to the principles outlined in the Declaration of Helsinki. The image sequences were imported into image processing software (Mimics 10.01; Materialise Inc., Leuven, Belgium) for 3D reconstruction. The reconstructed model was then smoothed using Geomagic Studio 12 (Geomagic Inc., Frankfurt, Germany).

#### Model Assembling and Fat Modeling

The FE eye model and orbital model were assembled following a previously reported method (see Fig. 2).^43^^,^^44^ First, eye protrusion (EP), lateral distance (LD), and superior distance (SD) were measured in the CT images, whereas orbital width (OW) and height (OH) were measured in the reconstructed orbital model. These measurements guided the rotation and translation of the eye model into the correct position relative to the orbital model. The space within the orbital socket not occupied by the eyeball was filled with fat.

#### Materials and Boundary Conditions

The choroid was assigned viscoelastic properties based on the experimental results described in the Viscoelastic Formulation section, with the viscoelastic parameters taken as the mean values from the two experimental directions. The vitreous and aqueous were assigned as a viscoelastic material and a liquid with specific viscosity, respectively, to approximate the physiological intraocular state. The material properties of the lens and ciliary body were kept consistent with those in the VT-WFU eye model. The cornea and sclera were modeled as linear elastic. The stiffness of these tissues was iteratively adjusted from initial values based on the work of Rossi et al.,^25^ with final values selected to match experimental data from the Delori BB impact experiment^45^ as detailed in Supplementary Tables S1 and S2. Table 2 lists the material properties, element types, and mesh numbers for all parts of the model. Given the high water content of most biological soft tissues, Poisson's ratios for all components were assigned values close to, but not exactly, the limiting value of 0.5.

**Table 1.** Mean ± Standard Deviation of Constitutive Parameters for Choroidal Tissue Specimens

	Fung Parameters					Prony (*n* = 3) Parameters						
Choroid Material	*c* (kPa)	*c_xx_* (−)	*c_xy_* (−)	*c_yy_* (−)	*R* ^2^	*g*_1_ (−)	*g*_2_ (−)	*g*_3_ (−)	*τ*_1_ (s)	*τ*_2_ (s)	*τ*_3_ (s)	*R* ^2^
Horizontal						0.18 ± 0.06	0.14 ± 0.03	0.21 ± 0.05	4.0 ± 1.4	74 ± 36	1040 ± 285	0.98
	22.3 ± 9.6	7.6 ± 1.3	5.7 ± 2.3	9.1 ± 2.5	0.97							
Vertical						0.19 ± 0.06	0.15 ± 0.05	0.21 ± 0.06	3.3 ± 1.1	81 ± 50	1099 ± 329	0.97

To ensure mechanical continuity within the eye, tie constraints were defined between neighboring tissues, such as between the choroid and the adjacent retina and sclera, and between the orbit and the surrounding fat. The BB was modeled as a rigid body with a diameter of 4.5 mm and a mass of 0.375 g, due to its much higher modulus compared with the ocular soft tissues. In the simulations, the BB was placed in direct contact with the corneal apex with no initial gap, and the impact direction was aligned with the corneal normal. The rim of the orbit was fixed to prevent its movement. The ocular tissues were meshed using hexahedral elements (C3D8R) with enhanced hourglass control enabled. The characteristic element size ranged from 0.15 mm (minimum) to 0.75 mm (maximum) across the eye model to balance numerical stability and computational efficiency in explicit dynamic simulations. The geometric features of the eye model are shown in Figure 2.

#### Mesh Convergence Analysis

To ensure numerical accuracy, a mesh convergence study was conducted for the choroid, sclera, and retina in the eye model. Three finite element meshes with different resolutions were generated for each tissue. Element sizes of 1.0, 0.3, and 0.2 mm were used for the choroid and sclera, and 1.0, 0.5, and 0.3 mm were used for the retina (meshes 1–3; Supplementary Fig. S1). Detailed mesh information is summarized in Supplementary Table S3. At a reference impact speed of 20 m/s, the peak von Mises stress in each tissue was extracted and compared across the 3 mesh models. The results showed that the differences in peak stress between Mesh 3 and Mesh 2 were less than 5%, indicating satisfactory mesh convergence. Based on these results, and considering both computational efficiency and accuracy, the mesh with an element size of 0.3 mm was selected for subsequent analyses of the choroid, sclera, and retina (Fig. 2C), containing 10,180, 42,108, and 10,180 elements, respectively.

#### Model Validation and Simulation

**Figure 3.**
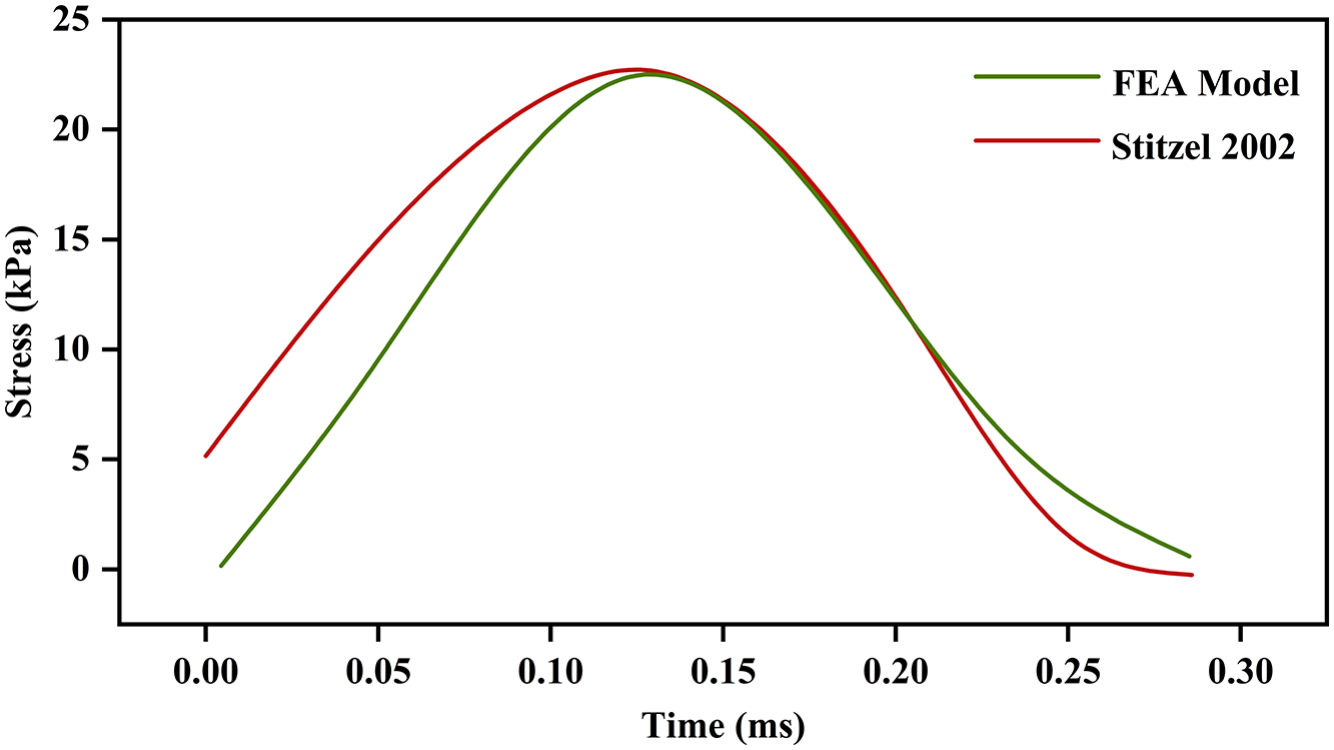
Corneal maximum principal stress time history for BB impact at 56 m/s, compared with Stitzel's data.

To validate the model, six matching simulations were conducted in ABAQUS 2020 using the VT-WFU eye model.^20^ The validation simulations were conducted using three distinct types of impactors: BB, foam object, and baseball, each selected to represent different levels of material hardness and impact characteristics. Two impact speeds were tested, categorized as low and high, to simulate different dynamic loading conditions. Correlation analyses were then performed using SPSS 24.0 (SPSS Inc.). Additionally, a representative validation curve for the 56 m/s impact case is provided (Fig. 3) to demonstrate the stress time-history. The results indicate that the current eye model captures the general dynamic response of the eye under blunt impact. Detailed information regarding the relevant loading conditions and matching simulation results is provided in the supplementary material. A parameter sensitivity analysis was conducted on the seven experimental viscoelastic parameters (*E*_0_, *g*_1_, *g*_2_, *g*_3_, τ_1_, τ_2_, and τ_3_) to assess their stability before the simulations. Each parameter was varied by ±20%. As summarized in Supplementary Table S4, these variations resulted in changes of less than 20% in the peak Mises stress within the choroid in all cases, thereby confirming the stability of the experimentally derived parameters.

Simulations of BB impacts on the corneal apex were conducted at various projectile speeds in ABAQUS 2020, with a duration of 5 ms. Impact speeds of 20, 40, and 60 m/s were selected to represent low-, moderate-, and high-severity ocular trauma conditions relevant to BB-related eye injuries.^46^ Additionally, one structural condition and one mechanical condition in the eye model were altered to investigate their effects on the choroid and surrounding tissues (sclera and retina). The mechanical responses of intraocular tissues, with and without the choroidal structure, were first evaluated under the three impact speeds. Subsequently, the choroid was assigned two different material properties - linear elasticity and viscoelasticity - within the eye model, and the mechanical response of the choroid was analyzed under the aforementioned loading conditions.

### Statistical Analysis

The normality of all experimental data was assessed using the Shapiro-Wilk test, and the results confirmed that the data were normally distributed. Therefore, paired-sample *t*-tests were performed to analyze statistical differences. Statistical analyses were conducted using SPSS version 24.0 (IBM Corp., Armonk, NY, USA). Results are presented as mean ± standard deviation; * indicates *P * < 0.05, ** indicates *P* < 0.01, *** indicates *P* < 0.001, and n.s. indicates no statistically significant difference between the data groups, that is, *P* > 0.05. For the simulation analysis, the Pearson correlation test was used to evaluate the accuracy of the model. A probability value (*P*) of less than 0.05 was considered statistically significant.

## Results

### Biaxial Tensile Test of Choroid

**Figure 4.**
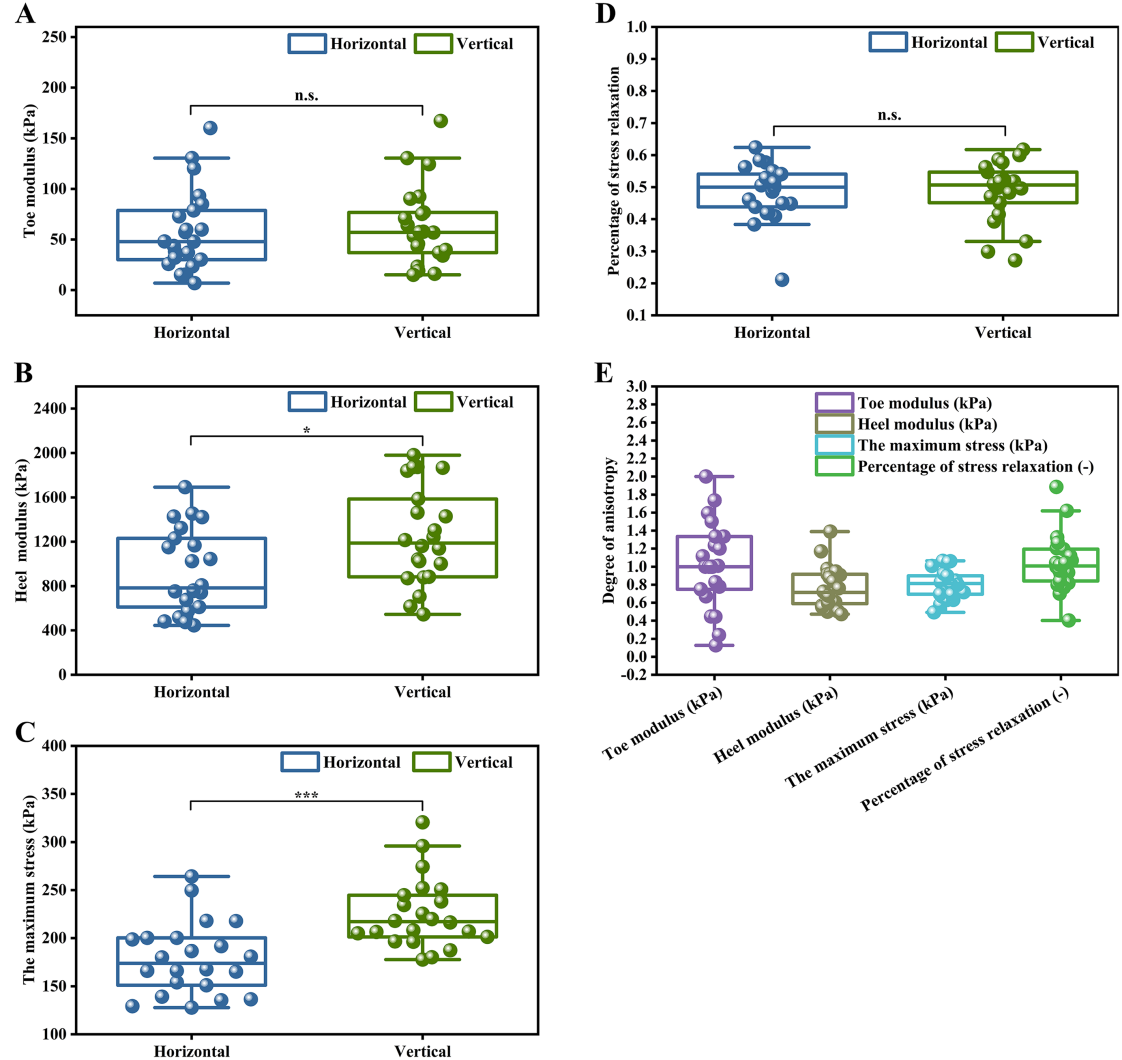
Orientation dependence of the elastic parameters of the choroid. (**A, B**) Toe modulus and heel modulus in the horizontal and vertical directions, respectively. (**C**) Maximum stress in the horizontal and vertical directions. (**D**) Percentage of stress relaxation in the horizontal and vertical directions. (**E**) Degree of anisotropy of these parameters.

As shown in Figure 4, for the choroid samples (*n* = 22), the toe modulus, heel modulus, maximum stress, and percentage of stress relaxation calculated in the horizontal direction were 58 ± 40 kPa, 933 ± 375 kPa, 178 ± 37 kPa, and 0.48 ± 0.09, respectively, whereas the corresponding values in the vertical direction were 63 ± 39 kPa, 1251 ± 438 kPa, 225 ± 36 kPa, and 0.49 ± 0.09, respectively. The difference in the average toe modulus between the vertical and horizontal directions is relatively small, showing a difference of 8% (*P* > 0.05). However, the average heel modulus in the vertical direction was significantly higher than that in the horizontal direction, showing a difference of 26% (*P* < 0.05). The average value of the maximum stress (nominal stress) calculated in the vertical direction was greater than that in the horizontal direction, and they differed by 21% (*P* < 0.001). The average value of percentage of stress relaxation in the vertical and horizontal directions showed no statistically significant difference (*P* *>* 0.05). Choroidal anisotropy degrees were 1.02 (95% confidence interval [CI] = 0.81–1.22) for toe modulus, 0.76 (95% CI = 0.66–0.87) for heel modulus, 0.80 (95% CI = 0.73–0.87) for maximum stress, and 1.04 (95% CI = 0.90–1.18) for percentage of stress relaxation.

**Figure 5.**
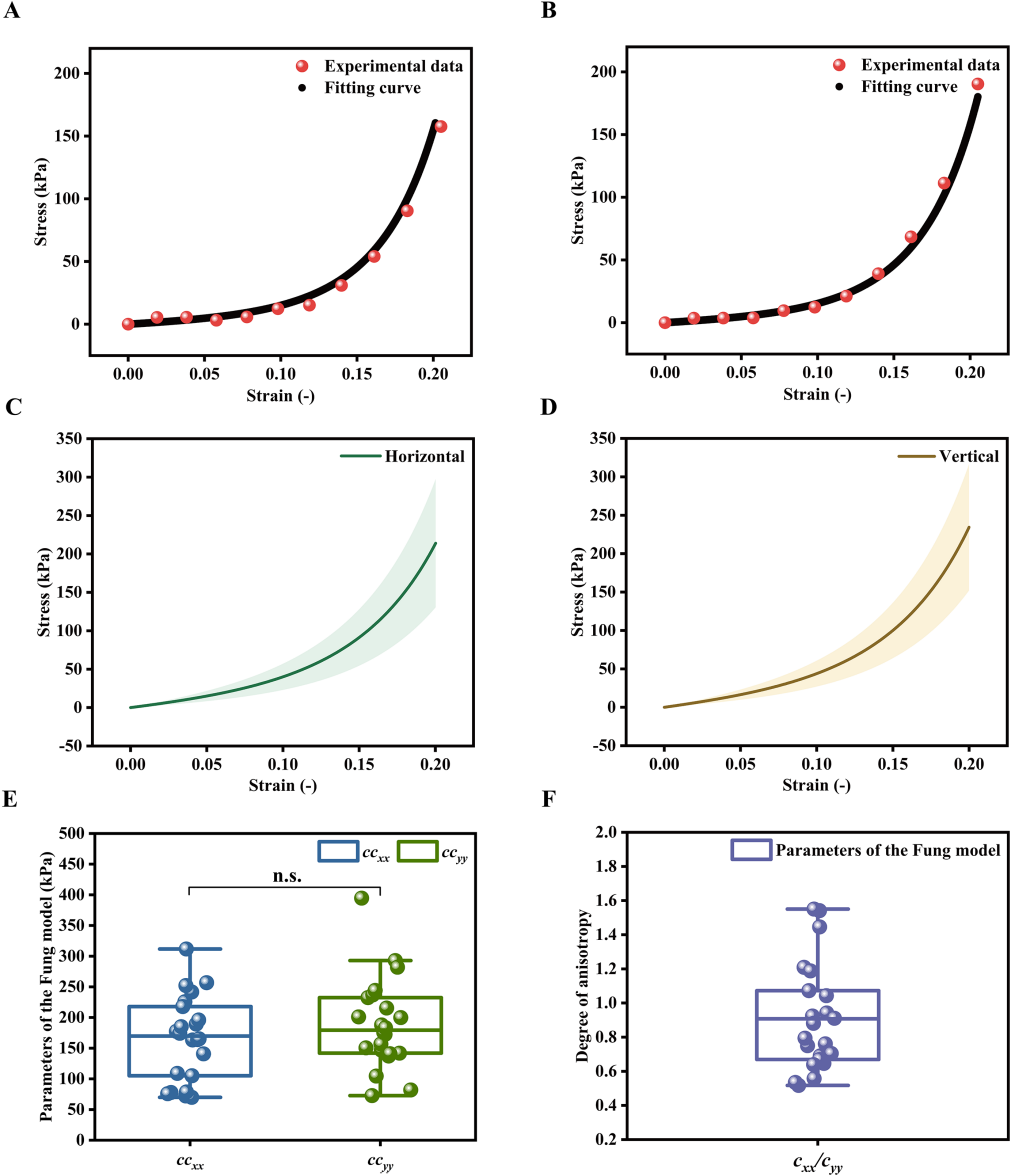
Tensile stress-strain responses of the choroid. (**A, B**) Representative stress-strain curves in the horizontal and vertical directions, respectively. (**C, D**) Average stress-strain responses of choroidal specimens in the horizontal and vertical directions, respectively. (**E**) Directional dependence of the Fung model parameters. (**F**) Average anisotropy of the choroidal specimens.

Representative stress-strain curves (Cauchy stress versus Green strain) for the porcine choroid under biaxial tensile loading are shown in Figures 5A and 5B. The curves indicate nonlinear behavior in both the horizontal and vertical directions over the range from the toe region to 20% strain. The fitted model agreed well with the experimental data, with *R*² values greater than 0.99. All material parameters obtained by fitting the Fung model using the Levenberg-Marquardt algorithm are listed in Table 1. All fitted parameter sets satisfied the convexity constraints, and the average goodness-of-fit value (*R*²) was greater than 0.96 across all specimens. These parameters may serve as input parameters for finite element models of the choroid. The average stress-strain curves in both directions are shown in Figures 5C and 5D. When averaged over all specimens, the stiffness products *cc_xx_* and *cc_yy_* were 166 ± 69 kPa and 189 ± 74 kPa, respectively (Fig. 5E). Based on the Fung model parameters, the choroidal specimens showed no specific pattern of anisotropy in equibiaxial tests, with a level of anisotropy (ratio of coefficients *cc_xx_*/*cc_yy_*) of 0.93 ± 0.3 (Fig. 5F).

**Table 2.** Material Parameters and Element Types in the Eye Model

Structure	Material Model	Density, kg/m^3^	Element Type	Number of Elements	Material Parameters
Cornea	Elastic	1,076	Hexahedron	6,180	Linear elastic (this work)
Sclera	Elastic	1,243	Hexahedron	42,108	Linear elastic (this work)
Choroid	Viscoelastic	1,000	Hexahedron	10,180	*E*_0_ = 1 MPa, *ν* = 0.47, *g*_1_ = 0.19, *g*_2_ = 0.15, *g*_3_ = 0.21, *τ*_1_ = 3.7, *τ*_2_ = 77, *τ*_3_ = 1070
Retina	Elastic	1,100	Hexahedron	10,180	*E* = 20 kPa, *ν* = 0.47
Aqueous	Fluid	1,000	Hexahedron	7,460	Shock EOS linear *C*_1_ = 1530 m/s, *s*_1_ = 2.1057; μ = 0.001 Pa·s
Lens	Elastic	1,078	Hexahedron	8,240	*E* = 6.88 MPa, *ν* = 0.47
Vitreous	Viscoelastic	1,050	Hexahedron	74,160	*G*_0_ = 10 Pa, *G*_∞_ = 0.3 Pa, *β* = 14.26 1/s, *ν* = 0.49
Ciliary	Elastic	1,600	Hexahedron	6,000	*E* = 11 MPa, *ν* = 0.47
Zonules	Elastic	1,000	Hexahedron	1,280	*E* = 357.78 MPa, *ν* = 0.47
Optic nerve	Elastic	1,243	Hexahedron	3,024	*E* = 5.5 MPa, *ν* = 0.47
BB	Rigid	7,860	Hexahedron	1,946	*E* = 200 GPa, *ν* = 0.3
Fat	Elastic	999	Tetrahedron	43,161	*E* = 0.047 MPa, *ν* = 0.49^28^
Orbit	Elastic	1,610	Tetrahedron	21,887	*E* = 14.5 GPa, *ν* = 0.35

*β*, viscoelastic decay constant; *C*_1_, speed of sound through the material; *E*, elastic modulus; *G*_0_, initial shear modulus; *G*_∞_, infinite shear modulus; ν, Poisson's ratio; *s*_1_, the coefficient related to the speed of the shocked material; μ, dynamic viscosity.

**Figure 6.**
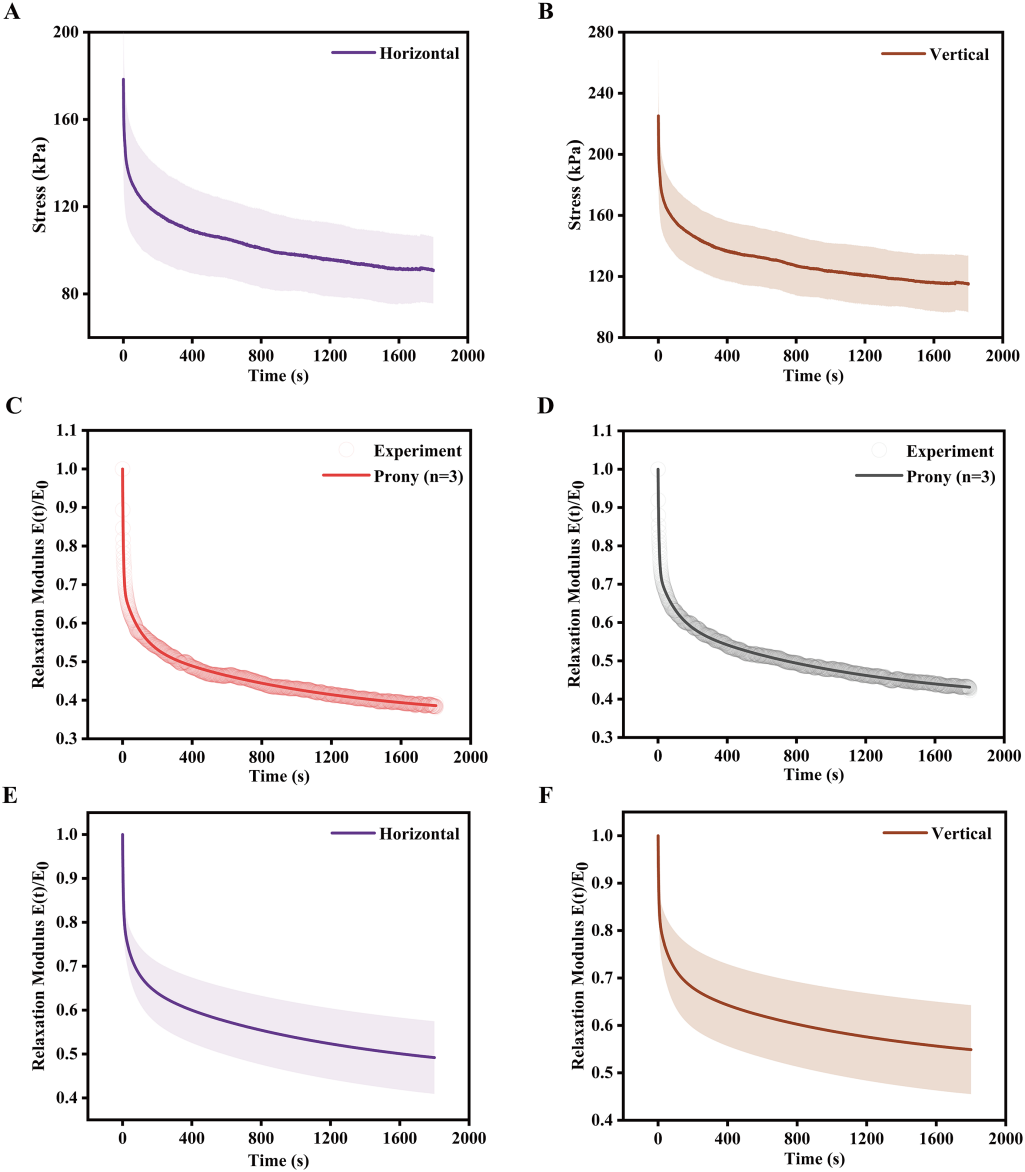
Tensile stress-relaxation behavior of the choroid. The *purple* and *brown curves* represent the mean experimental data, with *shaded areas* indicating the standard deviations. (**A, B**) Stress-relaxation responses in the horizontal (nasal-temporal) and vertical (superior-inferior) directions, respectively. (**C, D**) Prony series fitting obtained by least-squares regression in the horizontal and vertical directions, respectively. (**E, F**) Normalized relaxation modulus as a function of time in the horizontal and vertical directions, respectively.

In addition, the stress-relaxation behavior of the choroid in the horizontal and vertical directions is shown in Figures 6A and 6B. All the specimens of the choroid in both directions in the group (20% strain; *n* = 22) exhibited nonlinear stress relaxation behavior. The percentage of stress relaxation, used to characterize the viscoelastic behavior of the choroid, are presented in Figure 4D. This figure highlights the influence of specimen tensile direction on the viscoelastic response. The average value of the percentage of stress relaxation was almost the same in both directions (48% and 49%, *P* > 0.05) and the anisotropy index was approximately 1 (see Fig. 4E). Representative results of Prony series fitting to a choroidal specimen in the horizontal and vertical directions, obtained using least-squares regression, are shown in Figures 6C and 6D. Figures 6E and 6F show the normalized relaxation modulus versus time curves of the choroid in the two directions, respectively. A Prony series (*n* = 3) was fitted to the normalized relaxation modulus curves to derive viscoelastic material parameters for implementation in Abaqus. The resulting parameters are listed in Table 1 and can be used as inputs for finite element models.

### Numerical Simulation

**Figure 7.**
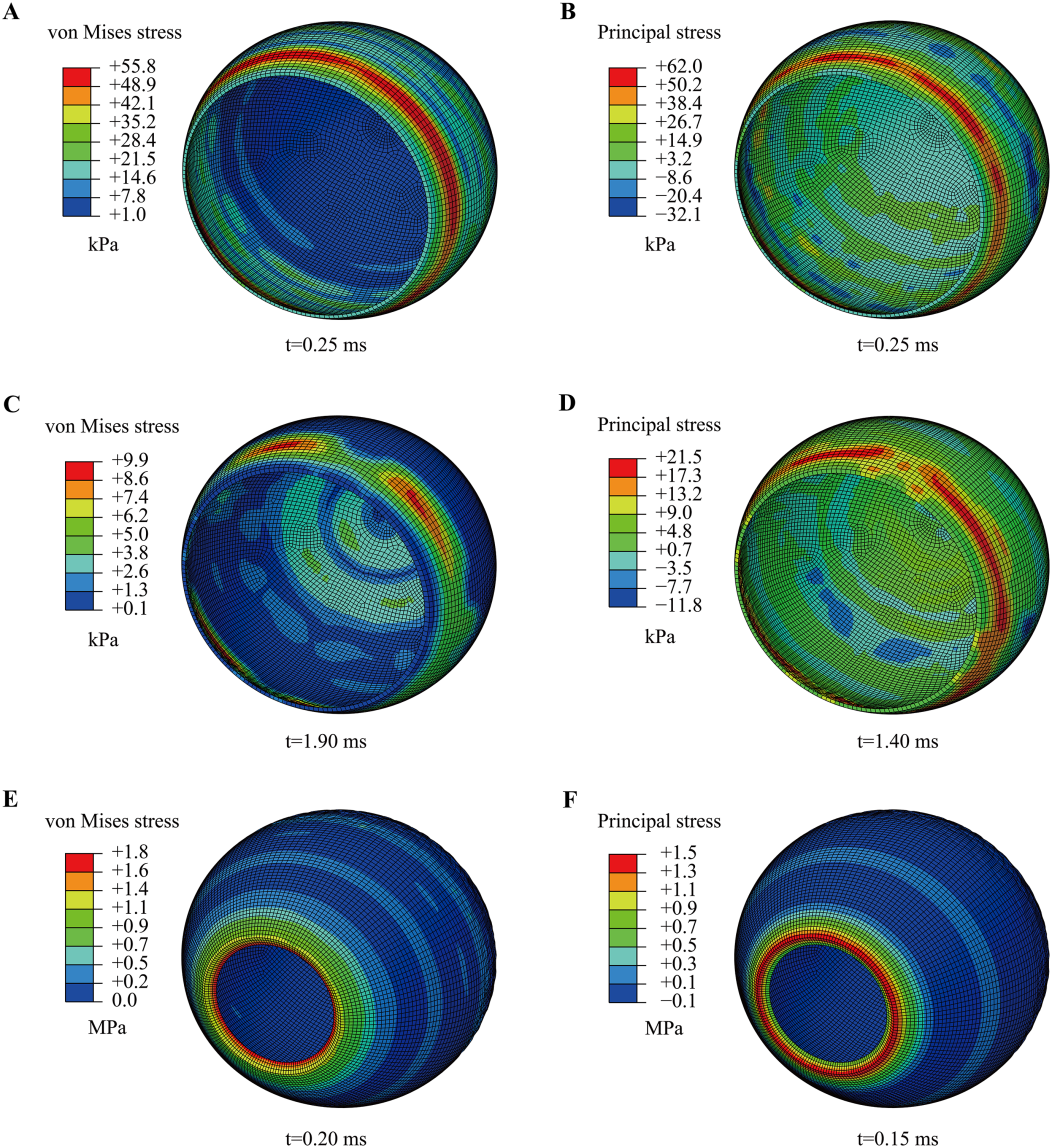
Stress distribution in the choroid and the adjacent retina and sclera. (**A**) Choroid von Mises stress; (**B**) Choroid principal stress; (**C**) Retina von Mises stress; (**D**) Retina principal stress; (**E**) Sclera von Mises stress; (**F**) Sclera principal stress.

The stress responses of the choroid and its neighboring tissues (the sclera and retina) were compared using two representative ocular trauma models at three impact speeds: one without the choroid and the other with a viscoelastic choroid. Figure 7 illustrates the stress distributions in the choroid, retina, and sclera in the eye model with a viscoelastic choroid at an impact speed of 20 m/s. Higher stress concentrations were observed in the adjacent anterior choroid and in the peripheral retina near the ora serrata (see Figs. 7A–D). In the sclera, stress concentrations consistently occurred at the corneoscleral limbus (see Figs. 7E, 7F). This pattern is likely attributable to the limbus serving as the transition zone between the cornea and sclera, where discontinuities in both material properties and geometry are present.^47^

**Figure 8.**
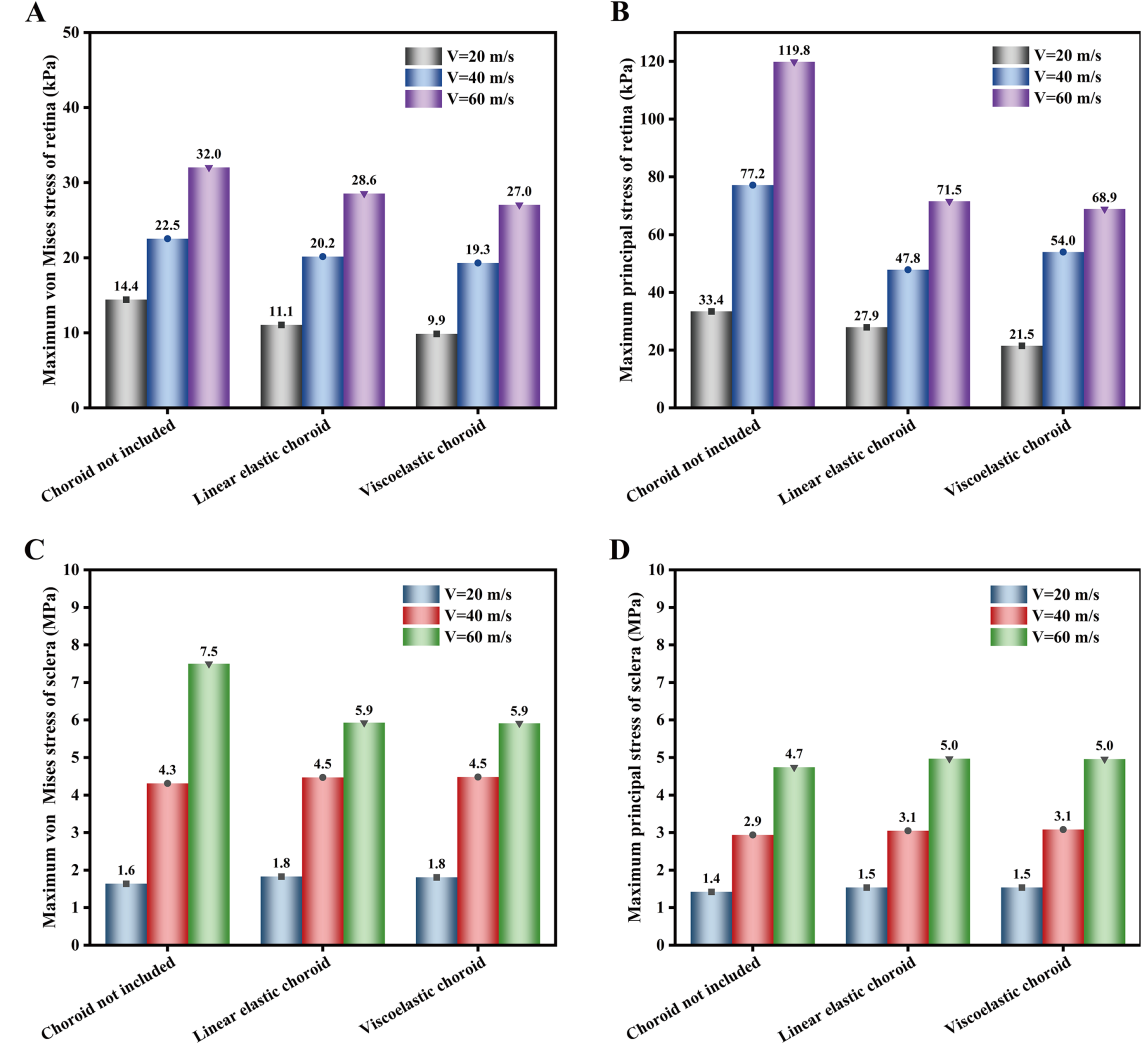
Bar charts showing the maximum stresses in the retina and sclera at different impact speeds for the three modeling approaches. (**A**) and (**B**) present comparisons of the maximum von Mises stress and maximum principal stress in the retina, respectively. (**C**) and (**D**) present comparisons of the maximum von Mises stress and maximum principal stress in the sclera, respectively.

Figure 8 illustrates the comparison of stresses in the retina and sclera across three ocular trauma models. As shown, the maximum stresses in the sclera and retina increased with increasing impact speed in all three models. Figures 8A and 8B show the maximum retinal stresses (both von Mises stress and principal stress) at three impact speeds. When the choroid was included in the ocular model, the maximum stress in the retina was considerably lower than that in the model without the choroid. Moreover, the effect of the choroid on retinal stress increased with impact speed. When the viscoelastic choroid was included in the ocular model, the maximum retinal stress differed from that in the model without the choroid. At a higher impact speed (*v* = 60 m/s), for example, the maximum von Mises stress and maximum principal stress in the retina were 32.0 kPa and 119.8 kPa, respectively, in the model without the choroid. In the model with the viscoelastic choroid, the corresponding values were 27.0 kPa and 68.9 kPa, respectively, representing reductions of 16% and 43%. Figures 8C and 8D show the maximum stresses in the sclera at three impact speeds. The maximum principal stress in the sclera was higher in the eye model including the choroid than in the model without the choroid. In addition, assigning either linear elastic or viscoelastic properties to the choroid had little effect on the maximum stress in the sclera. However, a maximum difference of over 20% (for the von Mises stress at *v* = 60 m/s) was still observed between the model without the choroid and the model with the viscoelastic choroid. Therefore, the effect of the viscoelastic choroid on scleral stress should not be overlooked.

**Figure 9.**
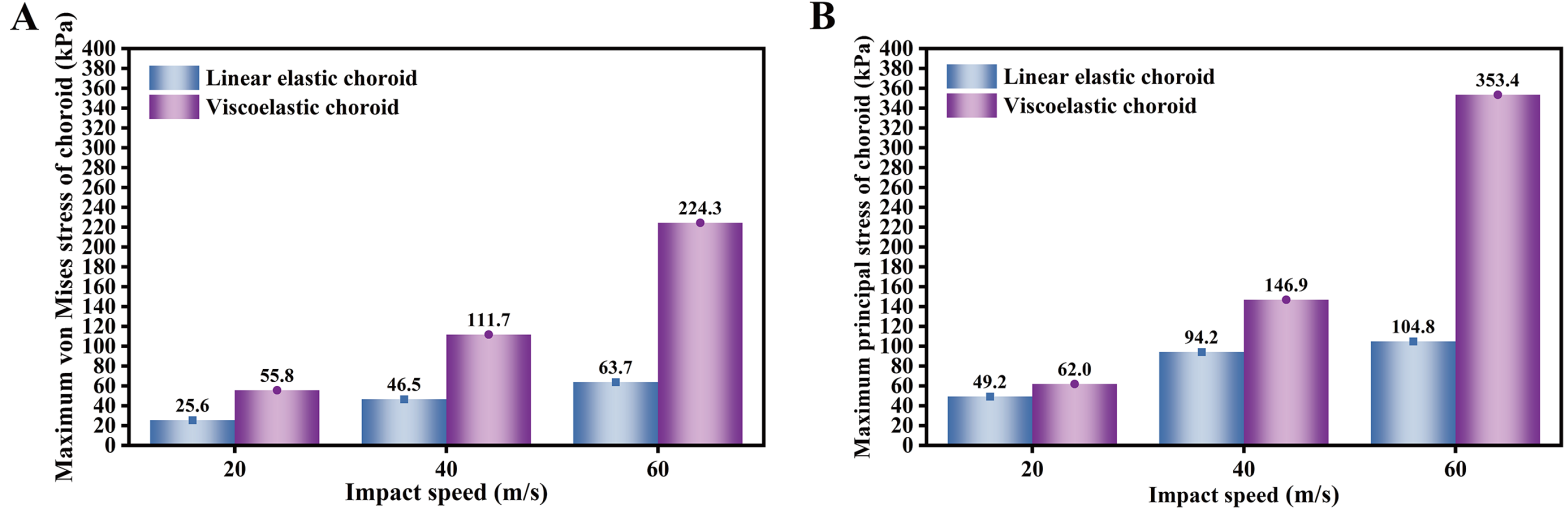
Comparison of the maximum stress in the choroid under three impact speeds with different assigned material properties. (**A**) Maximum von Mises stress; (**B**) Maximum principal stress.

Figures 9A and 9B show the effects of the choroid with two different material properties under the same loading conditions. When the choroid was modeled as linearly elastic, the maximum von Mises stresses were 25.6 kPa, 46.5 kPa, and 63.7 kPa at the 3 impact speeds, respectively. For the viscoelastic choroid, the corresponding values were 55.8 kPa, 111.7 kPa, and 224.3 kPa, representing differences of 54%, 58%, and 72%, respectively. In terms of maximum principal stress, the linearly elastic choroid exhibited values of 49.2 kPa, 94.2 kPa, and 104.8 kPa, whereas the viscoelastic choroid showed values of 62.0 kPa, 146.9 kPa, and 353.4 kPa, with differences of 21%, 36%, and 70%, respectively.

**Figure 10.**
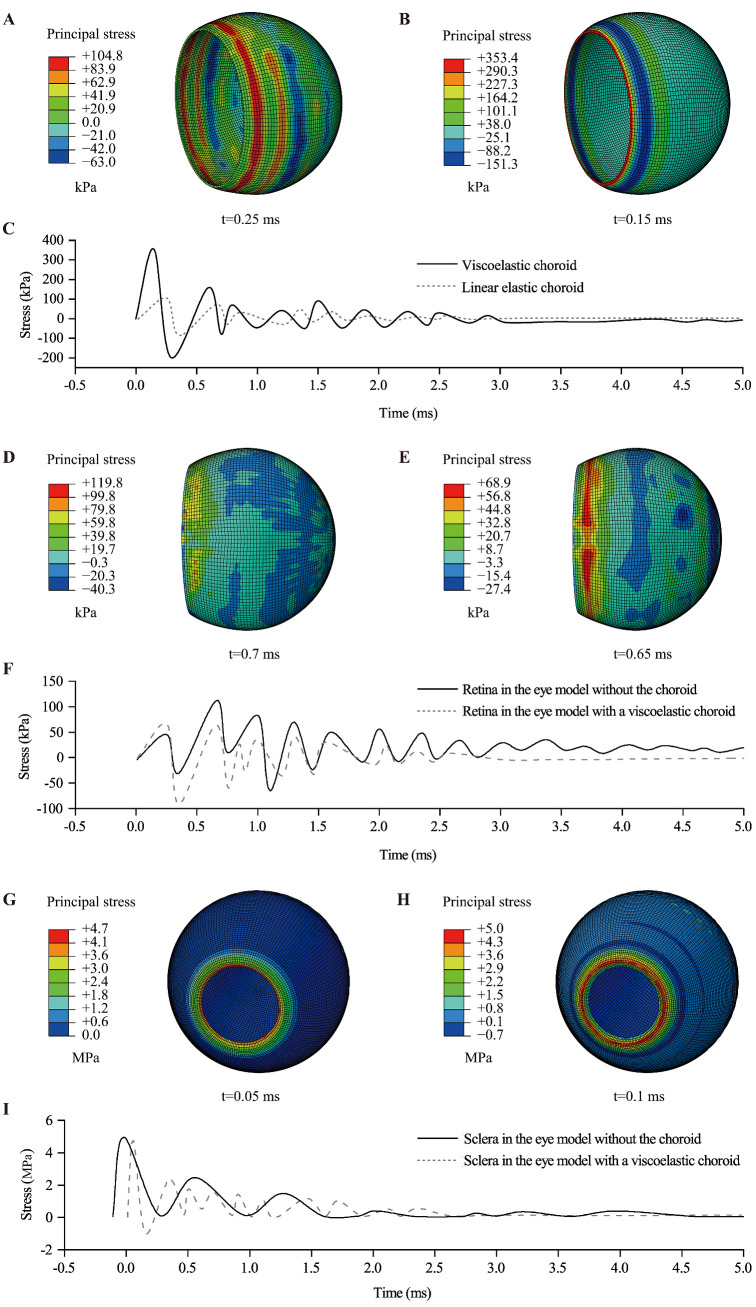
Stress distributions and stress-time history curves for the choroid (anterior region), retina (ora serrata region), and sclera (corneoscleral limbus region) at an impact speed of 60 m/s. (**A**) Linear elastic choroid. (**B**) Viscoelastic choroid. (**C**) Stress-time histories in the anterior choroid. (**D**) Retina in the eye model without the choroid. (**E**) Retina in the eye model with a viscoelastic choroid. (**F**) Stress-time histories in the retina near the ora serrata. (**G**) Sclera in the eye model without the choroid. (**H**) Sclera in the eye model with a viscoelastic choroid. (**I**) Stress-time histories at the corneoscleral limbus.

Principal stress was used to evaluate tissue damage in the posterior eye wall. Because elevated stress was consistently observed in the anterior choroid, the retina near the ora serrata, and the corneoscleral limbus (see Fig. 7), these regions were selected as the regions of interest (ROIs). As shown in Figure 10, substantial stress variations were induced in these regions by the transmitted force, followed by continuous attenuation. Compared with the anterior choroid and the corneoscleral limbus, the retina showed more pronounced stress oscillations. Notably, in the eye model without the choroid, the retinal principal stress reached its maximum value of 119.8 kPa at 0.7 ms.

Overall, the stress distributions in these tissues indicate that both the presence of the choroid and variations in its material properties affected the sclera and retina, with a greater effect on the retina. Furthermore, the differences in the mechanical responses of the linearly elastic and viscoelastic choroid to blunt trauma were also notable.

## Discussion

### Nonlinear Mechanical Properties of the Choroid

Overall, the experimental results enabled a comparison of mechanical parameters, including the toe modulus, of the porcine choroid along the nasal-temporal and superior-inferior directions. As shown in Figure 4A, the toe modulus of the porcine choroid exhibits no significant directional dependence (*P* > 0.05), with values of 58 ± 40 kPa and 63 ± 39 kPa in the 2 orthogonal directions. Although these values are higher than those reported previously (15 ± 22 kPa and 14 ± 22 kPa), this discrepancy is likely attributable to differences in experimental method, including specimen preparation and loading protocol. Importantly, the present finding of comparable toe modulus in both directions is consistent with the directional trend reported in the previous study.^11^ For the heel modulus, the measured values were 933 ± 375 kPa and 1251 ± 438 kPa in the 2 directions (see Fig. 4B), which are lower than the corresponding values reported previously (2222 ± 1440 kPa and 2542 ± 1462 kPa).^11^ This difference is most likely explained by the different tensile strain ranges adopted in the experiments. Our results show that the values in the vertical direction were significantly larger than those in the horizontal direction (*P* < 0.05). As shown in Figure 4C, the maximum stresses in the choroidal tissue differed significantly between the two directions (*P* < 0.001), with the stresses calculated in the vertical direction exceeding those in the horizontal direction. These findings align with the results reported by Zhang et al. from uniaxial testing of porcine choroidal soft tissue in two directions, respectively.^13^ In this study, the observed directional differences in heel modulus and maximum stress (vertical > horizontal) are mechanically plausible given that the choroid is a heterogeneous vascular-stromal tissue rather than a homogeneous continuum. Under large deformation (heel region), spatial heterogeneity in stromal constituents, vascular architecture, and mechanical coupling within the tissue may result in direction-dependent load sharing and constraint. Such effects can give rise to different tangent stiffness and peak stress responses along the superior-inferior and nasal-temporal directions.^8^

Additionally, this study explored the viscoelastic behavior of the choroid under biaxial loading in both the horizontal and the vertical directions. In the stress relaxation test involving a 20% deformation over 1800 seconds, the three-dimensional Wiechert model accurately captured the viscoelastic behavior of the tissues (*R*² > 0.97). This highlights that the quasi-linear viscoelastic formulation serves as an effective tool for characterizing the viscoelastic behavior of soft tissues. The model, constructed as a linear combination of multiple springs and dampers, provides a comprehensive depiction of the viscoelastic behavior of the choroid.

In conclusion, this study establishes a database of choroidal mechanical properties to support ocular trauma simulations. These data enable the development and validation of computational models for biomechanical analysis and the design of choroidal substitutes.^14^^,^^48^

### Computational Model of Ocular Trauma

To better characterize the biomechanical response of the eye to blunt impact, we developed a 3D FE model incorporating most major ocular structures and more realistic material properties. Using a stress-based criterion, we examined how the presence of the choroid and its material properties affect the mechanical responses of adjacent tissues and the choroid itself. Previous ocular trauma models often neglected the choroid or treated it as linearly elastic for computational convenience. However, as an anatomically indispensable and mechanically viscoelastic soft tissue, the choroid may play an important role in the transmission and redistribution of impact-induced stresses within the eye. Accordingly, incorporating the choroid more realistically into ocular trauma models is important for improving the biomechanical fidelity of the model and for achieving more reliable predictions of injury response.

The simulation results indicate that impact loading induces dynamic stresses within the intraocular tissues. In particular, the anterior choroid, the retina near the ora serrata, and the corneoscleral limbus may constitute biomechanically vulnerable regions, as these areas consistently showed relatively high stress levels (see Fig. 7). Wolter proposed the “coup-contrecoup” mechanism to explain ocular injuries resulting from blunt impact.^49^ Such injuries are most likely to occur at the interfaces between tissues with differing densities along the path of shockwave propagation. Previous studies have proposed that blunt ocular trauma is primarily caused by shockwave propagation.^23^^,^^26^ Our findings align with these conclusions. The retina and choroid are particularly vulnerable to shockwaves due to their low elastic modulus values. When the choroid is excluded from the eye model, the retina comes into direct contact with the sclera. This direct interface, coupled with the significant stiffness disparity between the retina and sclera, increases stress on the retina, rendering it highly susceptible to injury. However, when the choroid is incorporated into the model between the retina and sclera, it absorbs shock and promotes a rapid stress decline in the retina (see Figs. 8A, 8B). Under high strain-rate conditions, the instantaneous elastic modulus (*E*_0_) of the choroid becomes critical when its viscoelasticity is modeled. This creates a substantial stiffness gradient between the choroid and the retina, thereby elevating the stress levels in the retina. These findings highlight the significant effect of the choroid on neighboring tissues, particularly the retina, emphasizing the need for its proper consideration in ocular models. Additionally, simplifying the viscoelastic choroid as a linear-elastic material can have a substantial effect on its mechanical response under the same loading conditions (see Fig. 9). Therefore, caution must be exercised when simplifying intraocular tissue materials.

As shown in Figure 10, among the three tissues, the retina exhibited the most pronounced stress oscillations, suggesting that it may be more sensitive to dynamic stress transmission. In particular, the higher peak retinal principal stress observed in the model without the choroid suggests that the choroid may contribute to stress attenuation and redistribution within the eye. From a biomechanical perspective, this potential protective effect of the choroid may help reduce transient stress concentration in the retina and thereby lower the risk of tissue damage, especially in the peripheral retina near the ora serrata.

The present study provides reference data for studying ocular trauma, a leading cause of blindness. The accuracy of intraocular tissue interactions is heavily dependent on precise material properties when simulating blunt object impacts. To develop a realistic computer model of the eye, it is crucial to input appropriate material properties carefully, ensuring the reliability of the results. In future studies, this eye model could be integrated with a human skull model to simulate more extensive and commonly encountered ocular trauma scenarios, such as airbag deployment and diving-related injuries.

### Study Limitations

The inherent curvature of the eye presents challenges for biaxial testing of ocular tissues. In this study, curvature-related effects were mitigated by minimizing specimen dimensions while maintaining reliable mechanical measurements. Moreover, the high compliance and low stiffness of the choroid facilitate flattening during mounting, further reducing the influence of residual curvature. Nevertheless, complete planarization is not achievable, a limitation common to biaxial testing of curved soft tissues.

Although directional differences were observed in the heel modulus and maximum stress of the choroid, the overall anisotropy was moderate. Therefore, in the finite element simulations, the choroid was represented using averaged material parameters as an effective isotropic viscoelastic approximation. A fully anisotropic material model would require a large number of material parameters, many of which are currently difficult to obtain experimentally, and was therefore beyond the scope of the present study.

The cornea was modeled as a linear elastic, and the choroid was modeled as a linear viscoelastic material. These simplifications may affect the precision of the quantitative predictions, particularly under conditions of large deformation or transient loading. Nevertheless, they are not expected to alter the interpretation of the primary comparative trends. The choroid is a highly vascularized tissue,^8^ and therefore its ex vivo mechanical properties may differ from its in vivo behavior. Under physiological conditions, vascular perfusion and blood pressure can influence tissue stiffness and viscoelastic response. Previous studies have demonstrated that blood pressure-driven choroidal expansion and perfusion-dependent effects can alter model-predicted deformation and stress distributions in ocular tissues.^50^^,^^51^ These findings indicate that neglecting vascular perfusion and blood pressure may lead to an underestimation of the effective tissue stiffness and damping capacity under in vivo conditions.

To ensure numerical stability under dynamic loading conditions, a tied constraint assumption was adopted in the simulations as a simplifying approximation. Although this does not fully reflect the physiological choroid-retina interface, where attachment is limited and primarily occurs at penetrating vessels, it may enhance mechanical coupling and increase stress transfer between adjacent tissues. Moreover, given the relatively high stiffness of Bruch's membrane,^52^ incorporating it into the choroidal layer may increase the apparent tissue stiffness and result in slightly elevated predicted stresses in the choroidal region compared with explicitly layered models.

Despite these limitations, this study provides valuable insights into the biomechanical behavior of the choroid, offering reference data and a theoretical foundation for the analysis and assessment of ocular trauma.

## Conclusions

This study systematically characterized the mechanical properties of the porcine choroid through equibiaxial tensile testing and constitutive modeling, and incorporated experimentally derived viscoelastic data into an eye FE model for ocular trauma simulation. The results indicate that both omission of the choroid and simplification of the choroid as a linear elastic are inadequate for accurately capturing ocular mechanical responses. These findings highlight the importance of including a viscoelastic choroid in ocular models and provide a useful foundation for improving ocular trauma prediction and advancing the biomechanical understanding of the eye.

## Supplementary Material

Supplement 1

## References

[1] Négrel AD, Thylefors B. The global impact of eye injuries. *Ophthalmic Epidemiol*. 1998; 5(3): 143–169. 9805347 10.1076/opep.5.3.143.8364

[2] Kuhn F, Morris R, Witherspoon CD, Mann L. Epidemiology of blinding trauma in the United States Eye Injury Registry. *Ophthalmic Epidemiol*. 2006; 13(3): 209–216. 16854775 10.1080/09286580600665886

[3] Maurya RP, Srivastav T, Singh VP, Mishra CP, Al-Mujaini A. The epidemiology of ocular trauma in Northern India: a teaching hospital study. *Oman J Ophthalmol*. 2019; 12(2): 78–83. 31198291 10.4103/ojo.OJO_149_2018 PMC6561041

[4] Koval R, Teller J, Belkin M, Romem M, Yanko L, Savir H. The Israeli Ocular Injuries Study. A nationwide collaborative study. *Arch Ophthalmol*. 1988; 106(6): 776–780. 3370005 10.1001/archopht.1988.01060130846037

[5] Williams DF, Mieler WF, Williams GA. Posterior segment manifestations of ocular trauma. *Retina*. 1990; 10(suppl 1): S35–S44. 2191381 10.1097/00006982-199010001-00006

[6] Singh S, Saxena S. Unraveling the perplexities of choroidal rupture. *Indian J Ophthalmol*. 2023; 71(6): 2602–2603. 37322692 10.4103/IJO.IJO_326_23 PMC10417958

[7] Patel MM, Chee YE, Eliott D. Choroidal rupture: a review. *Int Ophthalmol Clin*. 2013; 53(4): 69–78. 24088934 10.1097/IIO.0b013e31829ced74

[8] Nickla DL, Wallman J. The multifunctional choroid. *Prog Retin Eye Res*. 2010; 29(2): 144–168. 20044062 10.1016/j.preteyeres.2009.12.002 PMC2913695

[9] Panozzo G, Mercanti A. Optical coherence tomography findings in myopic traction maculopathy. *Arch Ophthalmol*. 2004; 122(10): 1455–1460. 15477456 10.1001/archopht.122.10.1455

[10] Friedman E . The role of the atherosclerotic process in the pathogenesis of age-related macular degeneration. *Am J Ophthalmol*. 2000; 130(5): 658–663. 11078846 10.1016/s0002-9394(00)00643-7

[11] Chen K, Rowley AP, Weiland JD. Elastic properties of porcine ocular posterior soft tissues. *J Biomed Mater Res A*. 2010; 93(2): 634–645. 19591238 10.1002/jbm.a.32571

[12] Chen K, Rowley AP, Weiland JD, Humayun MS. Elastic properties of human posterior eye. *J Biomed Mater Res A*. 2014; 102(6): 2001–2007. 23852923 10.1002/jbm.a.34858

[13] Zhang ZH, Pan MX, Cai JT, Weiland JD, Chen K. Viscoelastic properties of the posterior eye of normal subjects, patients with age-related macular degeneration, and pigs. *J Biomed Mater Res A*. 2018; 106(8): 2151–2157. 29582545 10.1002/jbm.a.36417

[14] He Y, Guo Y, Wang JC, Lv WX, Li X, Chen K. The posterior eye with age-related macular degeneration has isotropic and nonlinear viscoelastic properties. *J Mech Behav Biomed Mater*. 2021; 114: 104207. 33307420 10.1016/j.jmbbm.2020.104207

[15] Das A, Frank RN, Zhang NL, Turczyn TJ. Ultrastructural localization of extracellular matrix components in human retinal vessels and Bruch's membrane. *Arch Ophthalmol*. 1990; 108(3): 421–429. 2310346 10.1001/archopht.1990.01070050119045

[16] Eilaghi A, Flanagan JG, Tertinegg I, Simmons CA, Brodland GW, Ethier CR. Biaxial mechanical testing of human sclera. *J Biomech*. 2010; 43(9): 1696–1701. 20399430 10.1016/j.jbiomech.2010.02.031

[17] Ferrara M, Lugano G, Sandinha MT, Kearns VR, Geraghty B, Steel DHW. Biomechanical properties of retina and choroid: a comprehensive review of techniques and translational relevance. *Eye (Lond)*. 2021; 35(7): 1818–1832. 33649576 10.1038/s41433-021-01437-w PMC8225810

[18] Geng X, Liu X, Wei W, et al. Mechanical evaluation of retinal damage associated with blunt craniomaxillofacial trauma: a simulation analysis. *Transl Vis Sci Technol*. 2018; 7(3): 16. 10.1167/tvst.7.3.16 PMC5991806 29888114

[19] Uchio E, Ohno S, Kudoh J, Aoki K, Kisielewicz LT. Simulation model of an eyeball based on finite element analysis on a supercomputer. *Br J Ophthalmol*. 1999; 83(10): 1106–1111. 10502567 10.1136/bjo.83.10.1106 PMC1722840

[20] Stitzel JD, Duma SM, Cormier JM, Herring IP. A nonlinear finite element model of the eye with experimental validation for the prediction of globe rupture. *Stapp Car Crash J*. 2002; 46: 81–102. 17096220 10.4271/2002-22-0005

[21] Liu XY, Wang LZ, Wang C, Fan J, Liu SY, Fan YB. Prediction of globe rupture caused by primary blast: a finite element analysis. *Comput Methods Biomech Biomed Engin*. 2015; 18(9): 1024–1029. 24661047 10.1080/10255842.2013.869317

[22] Cirovic S, Bhola RM, Hose DR, et al. Computer modelling study of the mechanism of optic nerve injury in blunt trauma. *Br J Ophthalmol*. 2006; 90(6): 778–783. 16421184 10.1136/bjo.2005.086538 PMC1860230

[23] Liu XY, Wang LZ, Du CF, Li DY, Fan YB. Mechanism of lens capsular rupture following blunt trauma: a finite element study. *Comput Methods Biomech Biomed Engin*. 2015; 18(8): 914–921. 25427212 10.1080/10255842.2014.975798

[24] Stitzel JD, Hansen GA, Herring IP, Duma SM. Blunt trauma of the aging eye: injury mechanisms and increasing lens stiffness. *Arch Ophthalmol*. 2005; 123(6): 789–794. 15955980 10.1001/archopht.123.6.789

[25] Rossi T, Boccassini B, Esposito L, et al. The pathogenesis of retinal damage in blunt eye trauma: finite element modeling. *Invest Ophthalmol Vis Sci*. 2011; 52(7): 3994–4002. 21330659 10.1167/iovs.10-6477

[26] Liu XY, Wang LZ, Wang C, Sun GY, Liu SY, Fan YB. Mechanism of traumatic retinal detachment in blunt impact: a finite element study. *J Biomech*. 2013; 46(7): 1321–1327. 23477788 10.1016/j.jbiomech.2013.02.006

[27] Rossi T, Boccassini B, Esposito L, et al. Primary blast injury to the eye and orbit: finite element modeling. *Invest Ophthalmol Vis Sci*. 2012; 53(13): 8057–8066. 23111614 10.1167/iovs.12-10591

[28] Power ED, Stitzel JD, Duma SM, Herring IP, West RL. Investigation of ocular injuries from high velocity objects in an automobile collision. *SAE Trans*. 2002; 111: 211–218.

[29] Watson R, Gray W, Sponsel WE, et al. Simulations of porcine eye exposure to primary blast insult. *Transl Vis Sci Technol*. 2015; 4(4): 8. 10.1167/tvst.4.4.8 PMC4555842 26336633

[30] Karimi A, Razaghi R, Biglari H, Sabbaghi H, Sera T, Kudo S. A comparative study to determine the optimal intravitreal injection angle to the eye: a computational fluid-structure interaction model. *Technol Health Care*. 2018; 26(3): 483–498. 29710740 10.3233/THC-160777

[31] Zhang CX, Lan YF, Guo HM, Gao ZP, Song J, Chen WY. The adhesion behavior of the retina. *Exp Eye Res*. 2023; 233: 109541. 37321365 10.1016/j.exer.2023.109541

[32] Luo RK, Peng LM, Wu XP, Mortel WJ. Mullins effect modelling and experiment for anti-vibration systems. *Polym Test*. 2014; 40: 304–312.

[33] Hatami-Marbini H, Pachenari M. Tensile viscoelastic properties of the sclera after glycosaminoglycan depletion. *Curr Eye Res*. 2021; 46(9): 1299–1308. 34325593 10.1080/02713683.2021.1874026 PMC8532128

[34] Sattari S, Mariano CA, Eskandari M. Biaxial mechanical properties of the bronchial tree: characterization of elasticity, extensibility, and energetics, including the effect of strain rate and preconditioning. *Acta Biomater*. 2023; 155: 410–422. 36328122 10.1016/j.actbio.2022.10.047

[35] Sigaeva T, Sattari S, Polzer S, Appoo JJ, Di Martino ES. Biomechanical properties of ascending aortic aneurysms: quantification of inter- and intra-patient variability. *J Biomech*. 2021; 125: 110542. 34237660 10.1016/j.jbiomech.2021.110542

[36] Chen Q, Wang Y, Li ZY. Re-examination of the mechanical anisotropy of porcine thoracic aorta by uniaxial tensile tests. *Biomed Eng Online*. 2016; 15(suppl 2): 167. 28155705 10.1186/s12938-016-0279-6 PMC5259859

[37] Fung YC . *Biomechanics: Mechanical Properties of Living Tissues*. 2nd ed. New York, NY: Springer-Verlag; 1993.

[38] Sacks MS, Sun W. Multiaxial mechanical behavior of biological materials. *Annu Rev Biomed Eng*. 2003; 5: 251–284. 12730082 10.1146/annurev.bioeng.5.011303.120714

[39] Guo HM, Lan YF, Gao ZP, et al. Interaction between eye movements and adhesion of extraocular muscles. *Acta Biomater*. 2024; 176: 304–320. 38296013 10.1016/j.actbio.2024.01.028

[40] Cheng H, Li M, Wu J, et al. A viscoelastic model for the rate effect in transfer printing. *J Appl Mech*. 2013; 80(4): 041019.

[41] Tapia-Romero MA, Dehonor-Gómez M, Lugo-Uribe LE. Prony series calculation for viscoelastic behavior modeling of structural adhesives from DMA data. *Ing Investig Tecnol*. 2020; 21(2): 1–10.

[42] Aboulatta A . *Finite element modelling of retinal detachment*. PhD thesis. Liverpool, UK: University of Liverpool; 2021.

[43] Weaver AA, Loftis KL, Duma SM, Stitzel JD. Biomechanical modeling of eye trauma for different orbit anthropometries. *J Biomech*. 2011; 44(7): 1296–1303. 21316057 10.1016/j.jbiomech.2011.01.004

[44] Weaver AA, Loftis KL, Tan JC, Duma SM, Stitzel JD. CT-based three-dimensional measurement of orbit and eye anthropometry. *Invest Ophthalmol Vis Sci*. 2010; 51(10): 4892–4897. 20463322 10.1167/iovs.10-5503

[45] Delori F, Pomerantzeff O, Cox MS. Deformation of the globe under high-speed impact: its relation to contusion injuries. *Invest Ophthalmol*. 1969; 8(3): 290–301. 5772720

[46] Takahashi R, Okamura K, Tsukahara-Kawamura T, et al. Finite element analysis of changes in tensile strain by airsoft gun impact on eye and deformation rate in eyes of various axial lengths. *Clin Ophthalmol*. 2020; 14: 1445–1450. 32546952 10.2147/OPTH.S249483 PMC7266397

[47] Karimi A, Razaghi R, Navidbakhsh M, Sera T, Kudo S. Computing the stresses and deformations of the human eye components due to a high explosive detonation using fluid-structure interaction model. *Injury*. 2016; 47(5): 1042–1050. 26861803 10.1016/j.injury.2016.01.030

[48] Djigo AD, Bérubé J, Landreville S, Proulx S. Characterization of a tissue-engineered choroid. *Acta Biomater*. 2019; 84: 305–316. 30476582 10.1016/j.actbio.2018.11.033

[49] Wolter JR . Coup-contrecoup mechanism of ocular injuries. *Am J Ophthalmol*. 1963; 56: 785–796. 14077183 10.1016/0002-9394(63)92943-x

[50] Jin Y, Wang X, Zhang L, et al. Modeling the origin of the ocular pulse and its impact on the optic nerve head. *Invest Ophthalmol Vis Sci*. 2018; 59(10): 3997–4010. 30098188 10.1167/iovs.17-23454

[51] Chuangsuwanich T, Hung PT, Wang X, et al. Morphometric, hemodynamic, and biomechanical factors influencing blood flow and oxygen concentration in the human lamina cribrosa. *Invest Ophthalmol Vis Sci*. 2020; 61(4): 3. 10.1167/iovs.61.4.3 PMC7401712 32271886

[52] Wang X, Teoh CKG, Chan ASY, Thangarajoo S, Jonas JB, Girard MJA. Biomechanical properties of Bruch's membrane-choroid complex and their influence on optic nerve head biomechanics. *Invest Ophthalmol Vis Sci*. 2018; 59(7): 2808–2817. 30029276 10.1167/iovs.17-22069

