# Supplementary material for: Biaxial Mechanical Behavior of the Choroid and Its Effect on Ocular Trauma Simulations
Source: Invest Ophthalmol Vis Sci. 2026 May 26;67(5):64. doi: 10.1167/iovs.67.5.64 (PMC13225304; doi:10.1167/iovs.67.5.64)
Supplement: Supplement 1 [file iovs-67-5-64_s001.docx]

**Supplement 1**

*Validation of the eye model*

*1. Methods*

To validate the current eye model, three types of blunt projectiles (BB, foam and baseball) were included in the six matched simulations to reproduce the simulations of VT-WFU eye model. Two different initial speeds (lower and higher) were applied in each type of projectiles. The contacts between the objects and the eye surface were defined to be “frictionless”. The projectiles impacting towards the corneal apex were simulated. Table S1 lists the details on the loading information.

Table S1^#^ loading information for the matched simulations

| **Simulation** | **Objects** | **Mass(g)** | **Diameter(mm)** | **Velocity(m/s)** | **Modulus(MPa)** |
| --- | --- | --- | --- | --- | --- |
| S1 | BB | 0.375 | 4.50 | 56.0 | 200,000 |
| S2 | BB | 0.375 | 4.50 | 92.0 | 200,000 |
| SF1 | Foam | 0.077 | 6.35 | 10.0 | 2.208 |
| SF2 | Foam | 0.077 | 6.35 | 30.0 | 2.208 |
| SB1 | Baseball | 146.5 | 76.10 | 34.4 | 12 |
| SB2 | Baseball | 146.5 | 76.10 | 41.2 | 12 |

*2. Results*

The comparison of the results included the peak stress, the peak strain and the peak deflection of the two models. Significant positive correlation was found when the Pearson correlation test was performed (R=0.986 for peak stresses and R=0.920 for peak strains). Table S2 lists the comparison of results between the current and the VT-WFU eye models.

Table S2^#^ Comparison of results between the current and the VT-WFU eye models in the matched simulations

| **Simulations** | **Peak Stress (MPa)** | | **Peak Strain** | |
| --- | --- | --- | --- | --- |
|  | **Current** | **VT-WFU** | **Current** | **VT-WFU** |
| S1 | 21.340 | 22.812 | 0.443 | 0.437 |
| S2 | 35.320 | 32.757 | 0.643 | 0.732 |
| SF1 | 3.085 | 3.180 | 0.109 | 0.009 |
| SF2 | 7.911 | 7.830 | 0.135 | 0.250 |
| SB1 | 21.87 | 22.153 | 0.416 | 0.309 |
| SB2 | 20.820 | 24.167 | 0.406 | 0.360 |
| Correlation | R=0.986 | | R=0.920 | |
| RMSE | 1.817 | | 0.086 | |

**Supplement 2**

*Mesh convergence analysis*


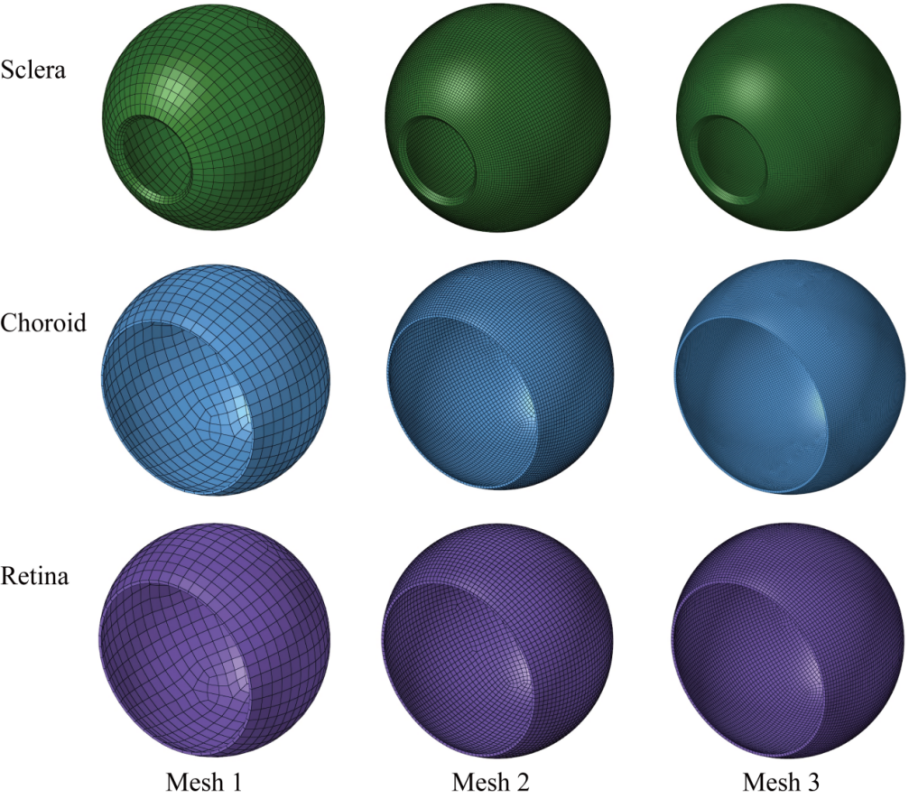


FIGURE S1 Three mesh models with different densities

Table S3^#^ Mesh convergence analysis of the model

| **Structure** | **Element size (mm)** | **Number of elements** | **Peak von Mises stress Stress** | **Variation** |
| --- | --- | --- | --- | --- |
| Choroid Mesh1 | 1 mm | 844 | 27.87 kPa | ＞5% |
| Choroid Mesh2 | 0.3 mm | 10180 | 34.53 kPa | (Reference) |
| Choroid Mesh3 | 0.2 mm | 33332 | 35.11 kPa | ＜5% |
| Sclera Mesh1 | 1 mm | 6528 | 1.17 MPa | ＞5% |
| Sclera Mesh2 | 0.3 mm | 42108 | 1.73 MPa | (Reference) |
| Sclera Mesh3 | 0.2 mm | 86076 | 1.76 MPa | ＜5% |
| Retina Mesh1 | 1 mm | 868 | 6.73 kPa | ＞5% |
| Retina Mesh2 | 0.5 mm | 5160 | 10.50 kPa |  |
| Retina Mesh3 | 0.3 mm | 10180 | 11.02 kPa | ＜5% (Reference) |

Table S4^#^ Sensitivity analysis of choroidal material parameters

| **Material parameters** | **Percentage change** | **Percentage change in peak Mises stress of the choroid** | **Percentage change in peak principal stress of the choroid** |
| --- | --- | --- | --- |
| E_0_-1 | +20% | ＜15% | ＜5% |
| g_1_-1 | +20% | ＜10% | ＜10% |
| g_2_-1 | +20% | ＜10% | ＜10% |
| g_3_-1 | +20% | ＜10% | ＜10% |
| *τ*_1_-1 | +20% | ＜10% | ＜10% |
| *τ*_2_-1 | +20% | ＜10% | ＜10% |
| *τ*_3_-1 | +20% | ＜10% | ＜10% |
| E_0_-2 | -20% | ＜15% | ≈20% |
| g_1_-2 | -20% | ＜10% | ＜10% |
| g_2_-2 | -20% | ＜10% | ＜10% |
| g_3_-2 | -20% | ＜10% | ＜10% |
| τ_1_-1 | -20% | ＜10% | ＜10% |
| τ_2_-1 | -20% | ＜10% | ＜10% |
| τ_3_-1 | -20% | ＜10% | ＜10% |
